# A Brain Motor Control Assessment (BMCA) Protocol for Upper Limb Function

**DOI:** 10.1371/journal.pone.0079483

**Published:** 2013-11-04

**Authors:** Maryam Zoghi, Mary Galea, David Morgan

**Affiliations:** Department of Medicine (Royal Melbourne Hospital), The University of Melbourne, Melbourne, Australia; University of Alberta, Canada

## Abstract

The Brain Motor Control Assessment (BMCA) protocol is a surface electromyography (sEMG)-based measure of motor output from central nervous system during a variety of reflex and voluntary motor tasks performed under strictly controlled conditions. The aim of this study was to evaluate the BMCA protocol for upper limb with the addition of shoulder voluntary tasks. The voluntary response index (VRI) was calculated from quantitative analysis of sEMG data during defined voluntary movement in neurologically intact people for comparison with that of patients after neurological injuries. The BMCA protocol included one bilateral and 4 unilateral voluntary tasks at different joints of both arms. The VRI, measured from 19 neurologically intact participants, comprises the total muscle activity recorded for the voluntary motor task (magnitude). The calculated similarity index (SI) for each phase of each task show the similarity of “the distribution of activity across the recorded muscles” for that task in this group off participants. Results: The VRI magnitude values from right and left sides for different tasks showed no significant difference (ANOVA: F_Side_: 0.09, P = 0.77). Therefore these values were pooled before calculating SI. SI values were higher for tasks against gravity: elbow flexion (0.99±0.03), wrist flexion with palm up (0.98±0.03) and wrist extension with palm down (0.97±0.07). On the other hand, the SI values were the lowest for bilateral shoulder abduction (0.84±0.08) and shoulder adduction (0.84±0.08). Conclusion: To validate this index for clinical use, serial studies on patients with neurological impairments should be performed. Tasks involving movement against gravity may be more suitable in future BMCAs.

## Introduction

The loss of upper limb function is one of the most significant and devastating losses after injuries to the central nervous system (CNS) (e.g. spinal cord injury, stroke, head injury). It has a severe impact on daily life and social activities, leading to subsequent dependence on others. The use of the upper extremities is critical in completing basic activities of daily living such as self-feeding, dressing, bathing and toileting as well as mobility needs (e.g. transfers from surface to surface and transitional movements) [1]. People with neurological dysfunction most frequently report that arm and hand function is the one of the main functions that they would like to be restored above all others [1]–[3].

The severity and extent of upper limb dysfunction are highly individualized even after similar lesions to the nervous system. Numerous measures are readily available to clinicians for the evaluation of these functions after neurological injuries [4] e.g. Action Research Arm Test [5], Box and Blocks Test [6], Chedoke Arm and Hand Activity Inventory, Jebsen-Taylor Hand Function Test [7], Nine-Hole Peg Test [8], the Wolf Motor Function Test [9], the Motor Activity Log [10] or Arm Activity Measure [11]. Many of these tests have been thoroughly evaluated for reliability and validity at multiple time points. The measures are either performance measures, where the clinician rates or times a series of upper limb actions that are performed by the patient, or self-report measures, where the clinician asks a series of questions about upper limb actions that are answered verbally by the patient or by proxy. In spite of the availability of all these assessment tools, none of them can provide clinicians with a complete picture of the impact of the injury to the nervous system in regards to upper limb functions. Although electrophysiological methods can complement the clinical evaluation by providing quantitative, objective data about the function of upper limb muscles; these techniques are not used routinely in clinical practice.

The aim of this study was to use neurophysiological recording techniques to provide comprehensive information about residual nervous system function post injury that cannot be determined using current clinical assessment techniques. These neurophysiological investigations can be used in the assessment of patients with neurological impairments in conjunction with clinical assessments to provide a more comprehensive picture of the injury in each patient. These evaluations would be very helpful in planning and measuring the results of therapeutic interventions in each case.

It has been suggested that surface electromyography (sEMG) recording during voluntary movement task performance might provide valuable information about the involved muscles and the supraspinal centres that control their activities [12]. sEMG is non-invasive, relatively easy to implement, and provides a quantitative measure of CNS output to muscles. It offers a view of CNS control that initiates, sequences, and coordinates muscle contractions to perform a movement. However, the electrophysiological measures are yet to be fully standardized and validated for clinical use, and there is a need for detailed guidelines. Assessment of voluntary limb movement control in neurological disorders should include a comparison with patterns produced by neurologically intact participants and should be able to demonstrate changes in control, e.g. due to interventions.

The Brain Motor Control Assessment Protocol (BMCA), which has been developed over the past decade, is a sEMG based measure of motor output from the CNS during a variety of reflex and voluntary motor tasks of the lower limb performed under strictly controlled conditions [12]. Quantification of motor activity recorded during this protocol has yielded repeatable, reliable, and appropriate descriptions of altered motor control [13], [14]. It has been shown that this protocol can add resolution to the clinical evaluation of residual supraspinal motor control, even in the absence of voluntary movement in patients with spinal cord injuries (SCI) [15] or head injuries [16]. These subclinical responses can be repeatable responses to reinforcement manoeuvres, responses to strong vibration that persist for at least 30 seconds [17] or the ability to volitionally suppress repeatable responses evoked by plantar surface stimulation in muscles innervated from below the SCI level [18].

Lee et al (2004) assessed the voluntary motor control in the lower limbs with the BMCA protocol in patients with SCI. They compared the multi-muscle activation patterns recorded from SCI participants with prototypes for standard simple motor tasks collected from neurologically intact participants [19], that are highly consistent when attempted under controlled conditions [12]. These voluntary response indices have shown high face validity [20] and reliability as a measure of SCI severity and treatment effects on volitional control [20]. Further, the indices were able to differentiate weak voluntary motor control from spasm activation in clinically motor complete SCI participants [21].

Despite the valuable information provided by the BMCA to quantify the motor control of lower limbs in SCI patients, the available data from the BMCA protocol for upper limb movements is limited. This study aimed to include shoulder complex voluntary tasks to this protocol and evaluate the movement pattern of these tasks in neurologically intact participants. Since we modified the voluntary task section of this protocol, we will only discuss the procedure and the results of this section of the BMCA protocol from a neurologically intact group of participants and then we will provide an example of using this voluntary task section in the assessment of a SCI patient over time.

## Methods

### Ethics statement

All participants gave their written informed consent before the assessments were carried out. All procedures used conformed with the Declaration of Helsinki, and the protocol was approved by the Human Research Ethics Committees at The University of Melbourne and Austin Health. Nineteen neurologically intact participants, 10 female and 9 male, were assessed. In addition, a spinal cord injured patient was chosen for comparison with prototypical patterns in neurologically intact participants. The SCI patient was a 29 years old male, one year post injury and classified as ASIA-A complete at level C4–C5. He was undertaking a rehabilitation program at the Royal Talbot Rehabilitation Centre at the time of assessments. The raw data for a voluntary task by this patient are presented for two occasions: baseline measurements recorded one year after injury, and the second assessment performed after 2 months of rehabilitation.

### Upper limb Brain Motor Control Assessment (BMCA) protocol

The upper limb BMCA protocol was modified based on adapted rules from the BMCA protocol for lower limbs[12]. The test was performed with participants lying supine, as this is the most comfortable position for neurologically intact participants and also for patients with neurologically impairments. Participants wore a singlet to allow access to the skin overlying upper limb muscles. At the beginning of the test, participants were asked to lie in the supine position on a plinth in a quiet and warm room with minimal distractions (e.g. noise, traffic).

The sEMG of 12 muscles (6 muscles from each side) were recorded throughout the experiment with self-adhesive pre-gelled disposable surface electrodes (Noraxon Dual electrodes, Scottsdale AZ, USA). Following skin preparation, pairs of sEMG electrodes, spaced 2 cm apart, were attached to the skin, oriented parallel to the long axis of the selected muscles. Due to the limited number of available EMG channels, 6 muscle groups were selected from each side. The muscles were selected so that all nerve roots from C_5_ to T_1_ were included. These were: pectoralis major (nerve supply: from C_5_ –T_1_ roots), deltoid (middle fibres, nerve supply from C_5_–C_6_ roots), biceps (nerve supply from C_5_–C_6_ roots), triceps (nerve supply from C_7_ root), wrist flexor muscle group (nerve supply from C_6_–C_7_ roots) and wrist extensor muscle group (nerve supply from C_7_ root). The skin under the electrodes was shaved and cleaned with alcohol. In each session the impedance between the 2 electrodes in the pair was less than 5 KΩ. EMG signals were amplified (x1000) (Wave Wireless EMG, Cometa, Milan, Italy) and then filtered (20–500 Hz) and digitised on-line (1 kHz sampling rate) using a PowerLab recording system (ADInstruments Ltd). All the EMG signals were hardware filtered (20–500 Hz) before any analysis.

As originally reported by Sherwood et al (1996)[12], the protocol included 7 stages: 1. Relaxation; 2. Reinforcement manoeuvres; 3. Voluntary tasks; 4. Passive movements; 5. Tendon-tap reflex responses; 6. Clonus; and 7. Vibration responses. In this paper we will only report the modified Voluntary Tasks and discuss the results.

The voluntary tasks included a bilateral task with two phases (shoulder abduction/adduction) and 4 unilateral tasks with two phases which were performed on both sides: shoulder abduction/adduction; elbow flexion/extension; wrist flexion/extension with palm up and wrist flexion/extension with palm down. All voluntary tasks in the BMCA protocol were cued by two 5 s tones for each phase with a brief pause between them, less than 1 second. Participants were asked to start the first phase at the tone and not to start the second phase until they heard the second tone. All tasks were repeated 3 times. After each trial the participants were given time to relax all the muscles before starting a new trial.

### Data reduction

Each voluntary motor task included 3 trials, each with two 5 s phases. The sEMG data was reduced to the root mean square (RMS) value over the 5 s interval, that was the basis for subsequent processing. Background activity was similarly measured from a 1 s window ending 1 s before the motor task. For each phase, the background was subtracted and the three trials averaged. This set of values, one for each muscle, comprised the response vector (RV) for each phase of a task. If the background signal exceeded the signal during a phase, a zero value was returned.

**Figure 1:**
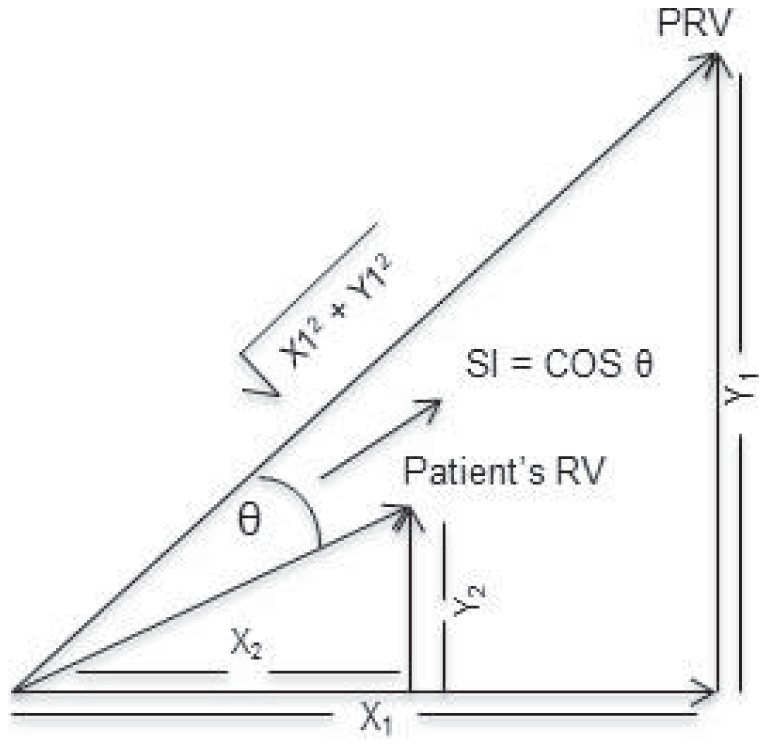
Representation of two muscle response vectors. SI is the cosine of the angle between the RV and PRV vectors (θ). This value compares the relative distribution across the set of muscles chosen for the task. A SI value of 1.0 means that the test participant's RV had an identical distribution of sEMG activity across muscles to the neurologically intact group PRV for that task. In the 2D case the ratio of activity in muscle X and muscle Y is the same. For two muscles (X and Y) the vectors are 2 dimensional as shown here. The vector sum is the response vector. A third muscle would be represented by a vector component perpendicular to the page. For more muscles, the formulae are extended, but visualisation in 3D space is no longer possible.

The response vector for each task was normalised, that is scaled by the magnitude of the vector, the square root of the sum of the squares of the vector components, ie activity of the selected muscles (Figure 1).

The selected muscles that were included for shoulder and elbow tasks in prototype calculations were deltoid, biceps, triceps and pectoralis major from both sides. The selected muscles that were included for wrist tasks in prototype calculations were deltoid, wrist flexors, wrist extensors and pectoralis major from both sides.

The normalization of the RV quantitatively describes the relative activity in each muscle during each phase of each movement. An average of normalized RVs (NRVs) across the nineteen neurologically intact participants was used to generate a prototype response vector (PRV) for each phase of each task in the protocol. Therefore, 18 PRVs were generated from the upper limb BMCA recordings in these participants. These values were used to calculate the similarity index (SI), which compares the relative distribution across the set of muscles chosen for the task.

The SI value, a numerical expression of the relationship of the NRV to the PRV, was computed as the cosine of the angle between the 2 vectors [19]. Note that this process means that, for these neurologically intact participants, they were included in the PRV with which they were compared, whereas usually the participant being tested would be excluded from the reference set. This was done to make the process used as close as possible to the process for patients, which will be compared with the average result of neurologically intact participants. For a large number of subjects the difference is expected to be small. Mean ± SD of the SI values across all participants were calculated for each phase of each task.

For unilateral tasks, the selected muscles were included from both sides. In neurologically intact participants, during a unilateral task, the contralateral prime movers remain relaxed. However, in neurologically impaired participants, the motor control pattern changes to adjust for weakness or paralysis of muscles. Therefore, these patients may show sEMG in muscles that would not normally be active during a specific task, e.g. antagonistic or contralateral muscles. Improvement in these patients can be assessed by comparing the similarity in the pattern of muscle activities of each task with that of a neurologically intact group. If patients are able to recruit the prime movers for a specific task and decrease unnecessary muscle activity, their SI scores should approximate neurologically intact values, indicating a positive effect of interventions. A value of 1.0 for the SI means that the test participant's RV had an identical pattern of sEMG activity across muscles to the neurologically intact group PRV for that task regardless of the EMG magnitude for those muscles. SI = 1 for a specific task means that corticospinal system was able to send commands to activate the right group of muscles for that task and keep the other muscles quiet even though those muscles are not strong enough to complete the task similar to healthy individuals for now.

ANOVA was used to assess the effect of side: right vs. left and gender: female vs. male on the magnitude of the RV for each phase of each unilateral task. ANOVA was also used to assess the effect of gender: female vs. male, side: right vs. left and tasks: 8 unilateral on the similarity index. Post-hoc tests (Bonferroni correction) were performed as required. Paired t-test were undertaken to compare the SI values of bilateral vs. unilateral tasks in shoulder joints and the SI values for the wrist flexion/extension task “against gravity” and “with gravity”.

## Results

**Figure 2:**
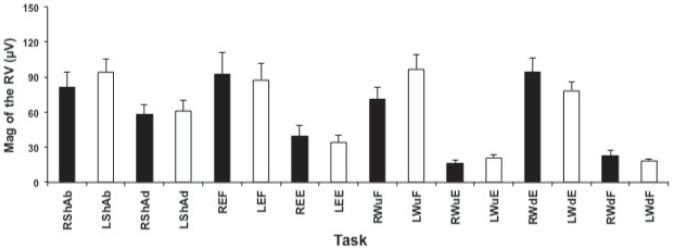
Mean ± SD of RV magnitude for each task in right and left side. Mean ± SD of RV magnitude for each task on right and left sides from 19 neurologically intact participants are presented in this figure. There was no significant difference between 2 sides for each task, P>0.05. R: right; L: left; ShAb: shoulder abduction; ShAd: shoulder adduction; EF: elbow flexion; EE: elbow extension; WFu: wrist flexion (palm up); WEu: wrist extension (palm up); WFd: wrist flexion (palm down); WEd: wrist extension (palm down).

**Figure 3:**
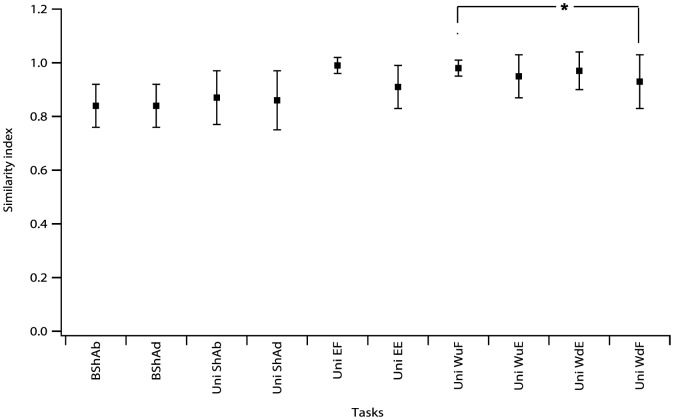
Mean ± SD of the SI scores of 19 neurologically intact participants. SI scores were calculated across 38 limbs for each phase of unilateral tasks. SI values were lowest for bilateral tasks (BShAb: 0.84±0.08; BShAd: 0.84±0.08) and were highest for the following unilateral tasks: elbow flexion (0.99±0.03), wrist flexion with palm up (0.98±0.03) and wrist extension with palm down (0.97±0.07). B: bilateral; Uni: unilateral; ShAb: shoulder abduction; ShAd: shoulder adduction; EF: elbow flexion; EE: elbow extension; WFu: wrist flexion (palm up); WEu: wrist extension (palm up); WFd: wrist flexion (palm down); WEd: wrist extension (palm down).

The results showed that the main effect of the “side” or the “gender” on the magnitudes of RV for each phase of each unilateral task was not significant in neurologically intact participants (F_Task_: 17.1, P<0.0001; F_Side_: 0.09, P = 0.77; F_Gender_: 1.64, P = 0.61) (Figure 2). Therefore the values were combined from both sides to calculate the PRV for each task. However, the main effect of the “Task” on SI was significant (F_Task_: 14, P<0.0001; but not the “side” (F_Side_: 0.24, P = 0.62) or the “gender” (F_Gender_: 3.5, P = 0.06). None of the interactions between these factors were significant. The lowest SI values (mean ± SD) were obtained for the bilateral task phases: (BShAb: 0.84±0.08; BShAd: 0.84±0.08) and the highest SI values were obtained for the elbow flexion (0.99±0.03) followed by unilateral tasks against gravity: wrist flexion with palm up (0.98±0.03) and wrist extension with palm down (0.97±0.07) (Figure 3).

SI values for bilateral shoulder task phases were not significantly different from the SI values of the unilateral shoulder task phases (α = 0.125, P>0.125).

The SI value for wrist flexion against gravity (WFu) was significantly higher than that when the task was performed with gravity (WFd) (*α* = 0.025, P<0.01).

On a per task basis, the best match to prototype is unilateral wrist extension with palm down (93% SI>0.95) followed by unilateral elbow flexion and wrist (Table 1). The worst match to prototype was bilateral shoulder abduction with no participants having an SI>0.95 followed by bilateral shoulder adduction (7%>0.95) and unilateral shoulder abduction with 13% SI>0.95).

**Table 1:** Distribution of matching prototypes on a per task basis.

SIs	Uni EF	Uni WFu	Uni WEd
**0.95–1.00**	92	89	91
**0.90–0.94**	5	5	6
**0.80–0.89**	3	5	0
**0.00–0.79**	0	0	3
**Total**	100	100	100

On a per task basis, the best match to prototype is unilateral elbow flexion (92%>0.95) followed by unilateral wrist extension with palm down and wrist flexion with palm up (91%>0.95 and 89%>0.95 respectively).

**Figure 4:**
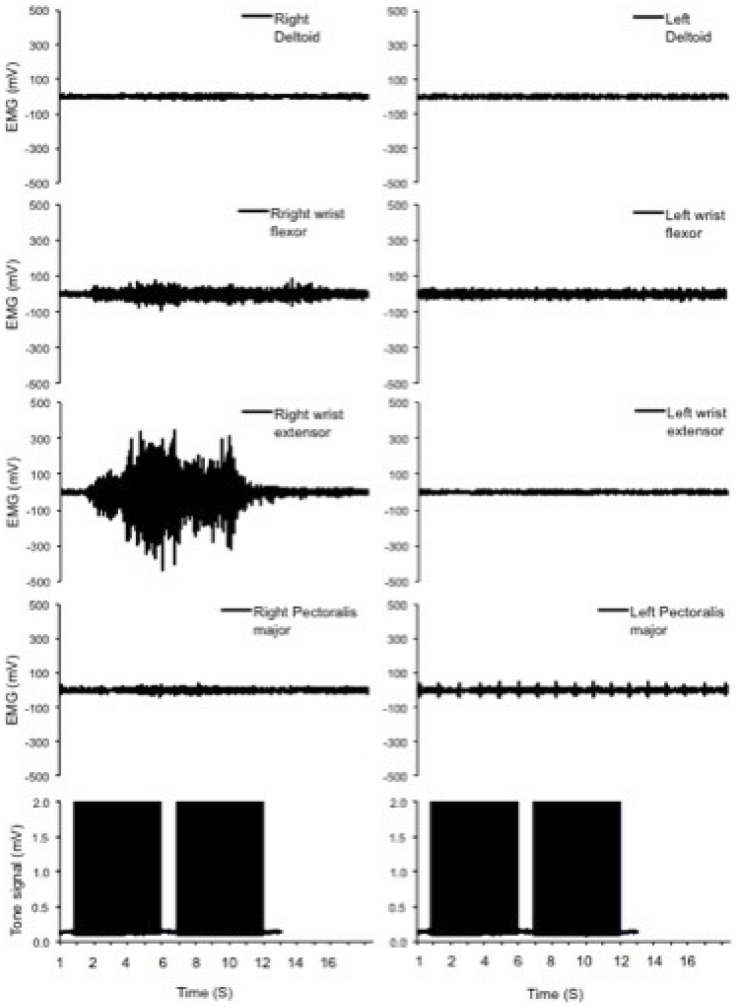
Representative data during wrist extension/flexion with palm down from a neurologically intact participant. The EMG activity of deltoid, wrist flexor muscles, wrist extensor muscles and pectoralis major muscles are presented from right and left sides during RWE/Fd. Each phase of this task was guided by a 5 sec tone (last panels). The right wrist extensor, as the prime mover, showed the greatest level of activity during this task. Some activity also can be seen in the right wrist flexors as one of the synergists throughout the movement. This participant managed to keep all the corresponding muscles relaxed on the left side during the task. On the trace of the left pectoralis major muscle, the detected heartbeats can be seen. RWE/Fd: wrist extension/flexion with palm down.

Figure 4 shows the representative data from one of the neurologically intact participants during right wrist extension/flexion with palm down figure (RWE/Fd) (wrist in a pronated position). The EMG activity of deltoid, wrist flexor muscles, wrist extensor muscles and pectoralis major muscles are presented from right and left sides during RWE/Fd. Each phase of this task was guided by a 5 sec tone (last panels). The right wrist extensor, as the prime mover, showed the major activity levels during this task. Some activity also can be seen in right wrist flexors as one of the synergists throughout the movement. This participant managed to keep all the corresponding muscles relaxed on the left side during the task. On the trace of the left pectoralis major muscle, the detected heartbeats can be seen.

**Figure 5:**
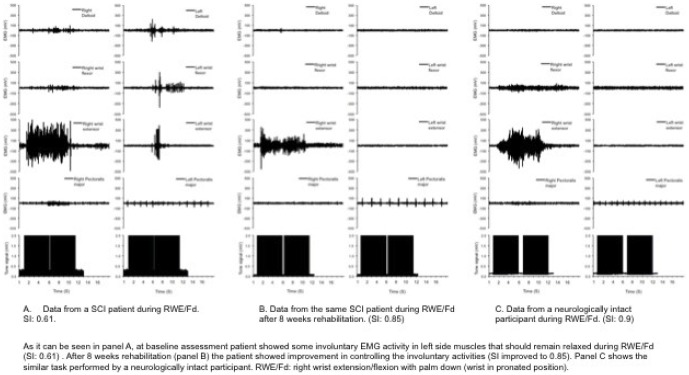
An example raw data from a SCI patient during wrist extension/flexion with palm down and similar data from a neurologically intact participant. Data from a SCI patient is presented in this Figure during RWE/Fd at two occasions (panel A: baseline; panel B: after 8 weeks rehabilitation). Panel A: at baseline assessment patient showed some involuntary EMG activity in left side muscles that should remain relaxed during RWE/Fd (SI: 0.61). Panel B: after 8 weeks rehabilitation the patient showed improvement in controlling the involuntary activity (SI improved to 0.85). Panel C shows the same task performed by a neurologically intact participant. RWE/Fd: wrist extension/flexion with palm down.

Data from a SCI patient is presented in Figure 5 during RWE/Fd on two occasions (panel A: baseline; panel B: after 8 weeks rehabilitation). As shown in Figure 4, panel A, at baseline assessment patient showed some involuntary EMG activity in muscles on the left side that should remain relaxed during RWE/Fd (SI: 0.61). After 8 weeks rehabilitation (panel B) the patient showed improvement in controlling this involuntary activity (SI improved to 0.85). Panel C shows the same task performed by a neurologically intact participant.

## Discussion

A bilateral, and two unilateral shoulder tasks with two phases and a unilateral wrist task with two phases were included in a BMCA protocol for upper limb functions and the SI values for all the tasks from neurologically intact people were measured. Although the BMCA protocol is easy to understand and follow, a prototype response may exhibit some internal variability. Due to biomechanical differences between upper and lower limbs and with more degrees of freedom in upper limb joints, voluntary motor tasks cannot be controlled for consistency as easily as lower limb joints.

Our preliminary study showed that wrist flexion/extension could not be assessed consistently with one two-phase task “wrist flexion/extension with palm up”. Participants showed some internal variability with phase 2, which was supposed to be an active wrist extension as well as different patterns of muscle activity during this phase. Some participants tended to relax their muscles and let gravity take their wrist back to resting position. Therefore, the protocol was lacking a proper evaluation of the pattern of muscle activity for active wrist extension. To overcome this problem, another task was added to the protocol and the instructions for wrist flexion/extension were changed accordingly. Two unilateral wrist tasks were defined: “wrist flexion/extension with palm up” and “wrist flexion/extension with palm down”. Active wrist flexion and extension against gravity were analysed in the first phase of each task respectively. In the second phase they were instructed to relax their muscles and let gravity take their wrist back to its resting position.

The RV magnitude for both wrist extension and flexion against gravity were significantly higher compared to when those movements were aided by gravity (P<0.0001). These findings are in agreement with previous studies. Virji-Babul et al (1994) examined the muscle activation patterns of single joint elbow movements made in the vertical plane [22]. They found that prime movers in both elbow flexion and extension made with gravity produced less EMG magnitude compared to when those movements made against gravity. They also reported that the pattern of muscle activations was influenced by the gravitational load [22]. Flexion and extension movements made with gravity were characterized by a reciprocally organized pattern of muscle activity in which phasic agonist activity was followed by phasic antagonist activity. However, flexion and extension movements made against gravity were characterized by early phasic antagonist activity occurring at about the same time as the initial agonist burst. This differential pattern of muscle activations is reflected in SI values reported in this study.

McKay et al. (2011) calculated the wrist extension RV from right and left upper trapezius, biceps brachi, triceps brachi, wrist extensors and wrist flexors muscles. They reported an SI value of 1.00 for this task. This group did not describe the position of hand during the task. Therefore it is not clear if the task was performed against gravity or with gravity. They also included the upper trapezius in their PRV calculations, a difference from this study.

The tasks that involved the shoulder complex showed the lowest SI values BShAb: 0.84±0.08; BShAd: 0.84±0.08). Lin et al. (2006) assessed the pattern of shoulder movements during four functional activities using SI calculations [23]. However, their data can not be compared to this study because the functional tasks and the muscles that they were recording from are quite different from those in the modified BMCA protocol used here.

One reason for the lower SI values with bilateral and unilateral shoulder movements is the unique structure and biomechanics of the shoulder complex. The shoulder complex consists of a series of articulations, numerous muscles and many ligaments, bursae and capsules, with more degrees of freedom than other joints in the upper limbs. It has been reported that the muscular stability of the glenohumeral joint is provided through the recruitment of primarily the rotator cuff, deltoid and long head of biceps [24] with muscles such as latissimus dorsi, teres major and pectoralis major being primarily responsible for movement [25]. In this study three muscles were selected from this complex (deltoid, biceps and pectoralis major) to assess the pattern of muscle activities during shoulder abduction/adduction. During the experiment, participants needed reminders to keep their arm in the horizontal plane throughout the whole range of bilateral and unilateral shoulder abduction/adduction. However, some participants were not able to fulfil the demands of this task completely based on the given instructions. Some participants tended to move gradually towards the scapular plane during shoulder abduction due to bulky shoulder muscles or variable flexibility in the ligaments or muscles around their shoulder complex. Although the consistency of movements in each plane can be affected by individual anatomical differences and variability in soft tissue flexibility, these participants were included in the prototype and SI calculations because these anatomical variations exist in the neurologically intact population and cannot be excluded. These SI values are calculated from the data of 38 limbs; however more participants might need to be recruited to decrease the effect of these variations in the final SI values. In addition the activity of more muscles from this complex needs to be included for calculation of the PRV for these movements. Since the SI values are higher for movements against gravity, shoulder flexion should be added to the protocol with relevant muscles to provide a better picture of motor outputs around the shoulder complex.

McKay et al. (2011) calculated SI values from 5 neurologically intact participants for 2 upper limb tasks (elbow flexion/extension and wrist extension) [26]. The selected muscles for calculating a PRV for these tasks were different from those in this study. They calculated the RVs for elbow flexion and extension from right and left upper trapezius, biceps brachii and triceps brachii muscles, and reported an SI value of 99% for elbow flexion which was similar to this study. However, they reported an SI value of 97% for elbow extension which was a higher value than found in this study (91%). Apart from the differences in the number of participants between the two studies, 5 versus 19 (38 limbs), the selected muscles in PRV were also different. In this study, deltoid and pectoralis major were included instead of upper trapezius in calculating the PRV for elbow flexion/extension. Upper trapezius is not active during elbow extension. However, pectoralis major is acting as a shoulder stabilizer during elbow extension and therefore, it can influence the PRV and SI value.

### Limitations of the study

The shoulder complex is one of the most sophisticated and complicated joints of the body. It has the greatest range of motion of any joint in the body. It consists of four joints and five linked bone groups which are related and work together. Six different movements (flexion/extension; abduction/adduction and internal rotation/external rotation) can be performed in three different planes (horizontal, vertical and scapular) in the shoulder complex. This complex requires coordinated activity of numerous muscles in set patterns to be able to produce these movements in everyday activities. However, in this study, two movements (abduction/adduction) in one plane (horizontal) were assessed. To better understand the normal pattern of muscular activation around the shoulder complex, consideration should be given to the inclusion of more movements in different planes in the BMCA protocol e.g. shoulder flexion in supine position in two different planes (sagittal vs. scapular plane which is 30° to 45° from the coronal plane). It has been shown that rotator cuff muscles can be recruited more easily in this plane compared to the sagittal plane.

To validate this index for clinical use, serial studies using patients with neurological impairments should be performed.

### Conclusion

This paper proposed the use of BMCA as an objective analysis method for assessing CNS motor control of upper limb voluntary movements and detecting changes during recovery after CNS injuries and post interventions. Normative patterns for one bilateral and four unilateral voluntary tasks of both arms were calculated. The comparison to the average patterns recorded from neurologically intact participants decreases the impact of the cross-subject variability by calculating a similarity index separately from the magnitude value. However, to employ this analysis in other voluntary tasks, normative patterns would need to be recorded and prototype calculations prepared for those tasks.
